# Reusable Multielectrode Array Technique for Electroencephalography in Awake Freely Moving Mice

**DOI:** 10.3389/fnint.2018.00053

**Published:** 2018-10-26

**Authors:** Carrie R. Jonak, Jonathan W. Lovelace, Iryna M. Ethell, Khaleel A. Razak, Devin K. Binder

**Affiliations:** ^1^Division of Biomedical Sciences, School of Medicine, University of California, Riverside, Riverside, CA, United States; ^2^Department of Psychology, University of California, Riverside, Riverside, CA, United States; ^3^Neuroscience Graduate Program, University of California, Riverside, Riverside, CA, United States

**Keywords:** multielectrode, array, electroencephalography, event-related potentials, biomarkers

## Abstract

Translational comparison of rodent models of neurological and neuropsychiatric diseases to human electroencephalography (EEG) biomarkers in these conditions will require multisite rodent EEG on the skull surface, rather than local area electrocorticography (ECoG) or multisite local field potential (LFP) recording. We have developed a technique for planar multielectrode array (MEA) implantation on the mouse skull surface, which enables multisite EEG in awake and freely moving mice and reusability of the MEA probes. With this method, we reliably obtain 30-channel low-noise EEG from awake mice. Baseline and stimulus-evoked EEG recordings can be readily obtained and analyzed. For example, we have demonstrated EEG responses to auditory stimuli. Broadband noise elicits reliable 30-channel auditory event-related potentials (ERPs), and chirp stimuli induce phase-locked EEG responses just as in human sound presentation paradigms. This method is unique in achieving chronic implantation of novel MEA technology onto the mouse skull surface for chronic multisite EEG recordings. Furthermore, we demonstrate a reliable method for reusing MEA probes for multiple serial implantations without loss of EEG quality. This skull surface MEA methodology can be used to obtain simultaneous multisite EEG recordings and to test EEG biomarkers in diverse mouse models of human neurological and neuropsychiatric diseases. Reusability of the MEA probes makes it more cost-effective to deploy this system for various studies.

## Introduction

One primary goal of modern neuroscience is to develop techniques for simultaneous monitoring of large areas of brain in awake and freely moving animals (Nicolelis et al., 1997; Buzsáki, 2004; Miyakawa et al., 2012; Alivisatos et al., 2013; Berényi et al., 2014; Mendoza et al., 2016). Such techniques have ranged in scale from monitoring individual neurons and local field potentials (LFPs) with microelectrodes such as “silicon probes” (Berényi et al., 2014) to larger-scale recordings such as grid electrodes for electrocorticography (ECoG) (Ledochowitsch et al., 2015; Milikovsky et al., 2017).

However, for translational comparison of rodent models to human EEG data, neither multisite LFP recording nor cortical surface recording (electrocorticography) is appropriate. Rather, many human neuropsychiatric and neurodevelopmental disorders have specific associated EEG abnormalities (recorded at the scalp) (Light and Swerdlow, 2015; Ethridge et al., 2017), and the ideal rodent model would be the implementation of multisite skull surface EEG recordings. For example, Fragile X syndrome (FXS), the most common inherited cause of intellectual disability and autism (Hagerman et al., 2017), is associated with specific EEG changes in human subjects. Augmentation of the auditory N1 potential (Castrén et al., 2003), alterations in brain functional connectivity (van der Molen et al., 2014), reduced habituation of auditory evoked potentials (Ethridge et al., 2016), abnormal resting state EEG (Van der Molen and Van der Molen, 2013) including increased resting gamma band power and spatial spreading of gamma band activity (Wang et al., 2017) and decreased phase locking to an auditory chirp stimulus (Ethridge et al., 2017) have all been reported in human subjects with FXS. While certain electrophysiological abnormalities such as the reduced habituation of N1 amplitude have been reproduced in studies of *Fragile X Mental Retardation (Fmr1) gene* knockout mice with 2-channel EEG recordings (Lovelace et al., 2016), analysis of the full spectrum of abnormalities seen in human EEG recordings would require multisite EEG measurements in an awake animal model.

To this end, we have developed and now report a reproducible method for *in vivo* multielectrode array (MEA) implantation on the surface of the mouse skull which can be used for baseline and stimulus-evoked EEG acquisition and analysis in awake, freely moving mice. We demonstrate MEA probe handling, implantation technique, and reusability for the first time. We further demonstrate the applicability of this new method to reliably obtaining multisite EEG recordings both at baseline and in response to auditory stimuli. This protocol can be applicable to diverse mouse models of human neuropsychiatric disease in which multisite EEG analysis is desired, and can be used to monitor EEG biomarkers in pre-clinical studies associated with potential treatments as in humans (Schneider et al., 2013).

## Materials and methods

### Animals

Jackson Laboratory C57Bl/6 and FVB male mice, 12 weeks old, were housed under a 12-h light and 12-h dark cycle with *ad libitum* access to food and water. All experiments were approved by the University of California, Riverside Institutional Animal Care and Use Committee (IACUC) and were conducted in accordance with the National Institutes of Health guidelines.

### MEA probes and EEG acquisition hardware

**Figure 1:**
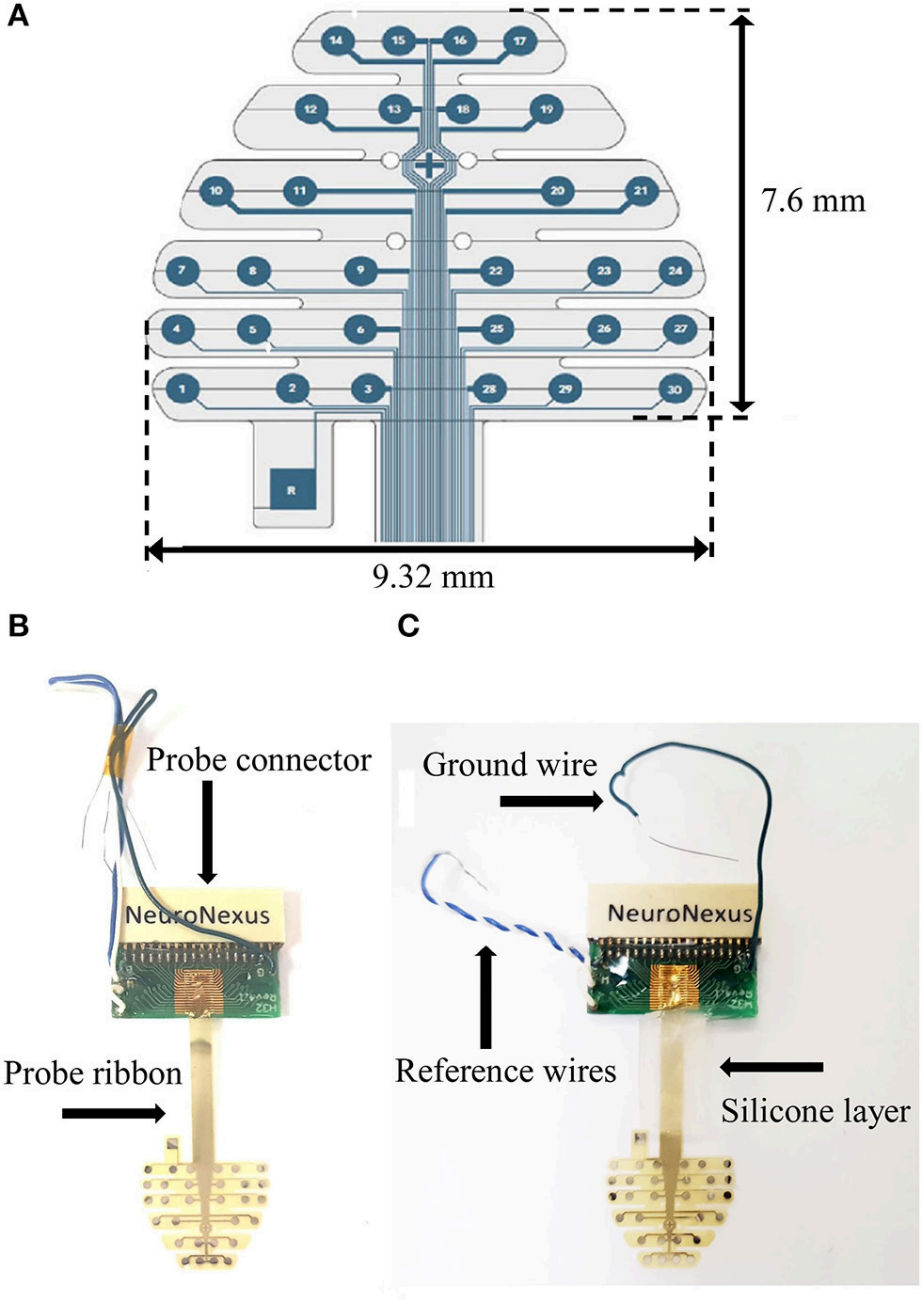
**(A)** MEA schematic (with dimensions). **(B)** Stock MEA probe. **(C)**. Silicone application to MEA ribbon.

A 30-contact multielectrode array (MEA) probe (NeuroNexus, Mouse EEG_v2-H32) (Figure 1A) was employed in these studies. This MEA probe consists of a planar array of platinum electrodes with array thickness 20 μm. The probe essentially lies down as a “sheet” over the surface of the mouse skull as described and shown below.

It is subsequently connected to a headstage (NeuroNexus, SmartLink32), which allows digitization of the neural signal at the head of the animal, minimizing noise and movement artifact. For digital EEG signal acquisition, we used the NeuroNexus Smartbox™, which has 30 kHz sampling per channel, 16-bit A/D converter, records up to 256 channels simultaneously, streams raw data to disk allowing flexibility for post-processing, and reads data into MATLAB or exports to other programs.

### Multielectrode array (MEA) preparation

1. Prior to the implantation there are several modifications to the stock probe (Figure 1B) that we have found to aid in reusability.
2. Twist the white and blue reference wires together and add a layer of silicone (Factor II, A-100) to the probe ribbon (Figure 1C). Let dry overnight.
3. Position reference wires and hold together with electrical tape.

### Implantation procedure

**Figure 2:**
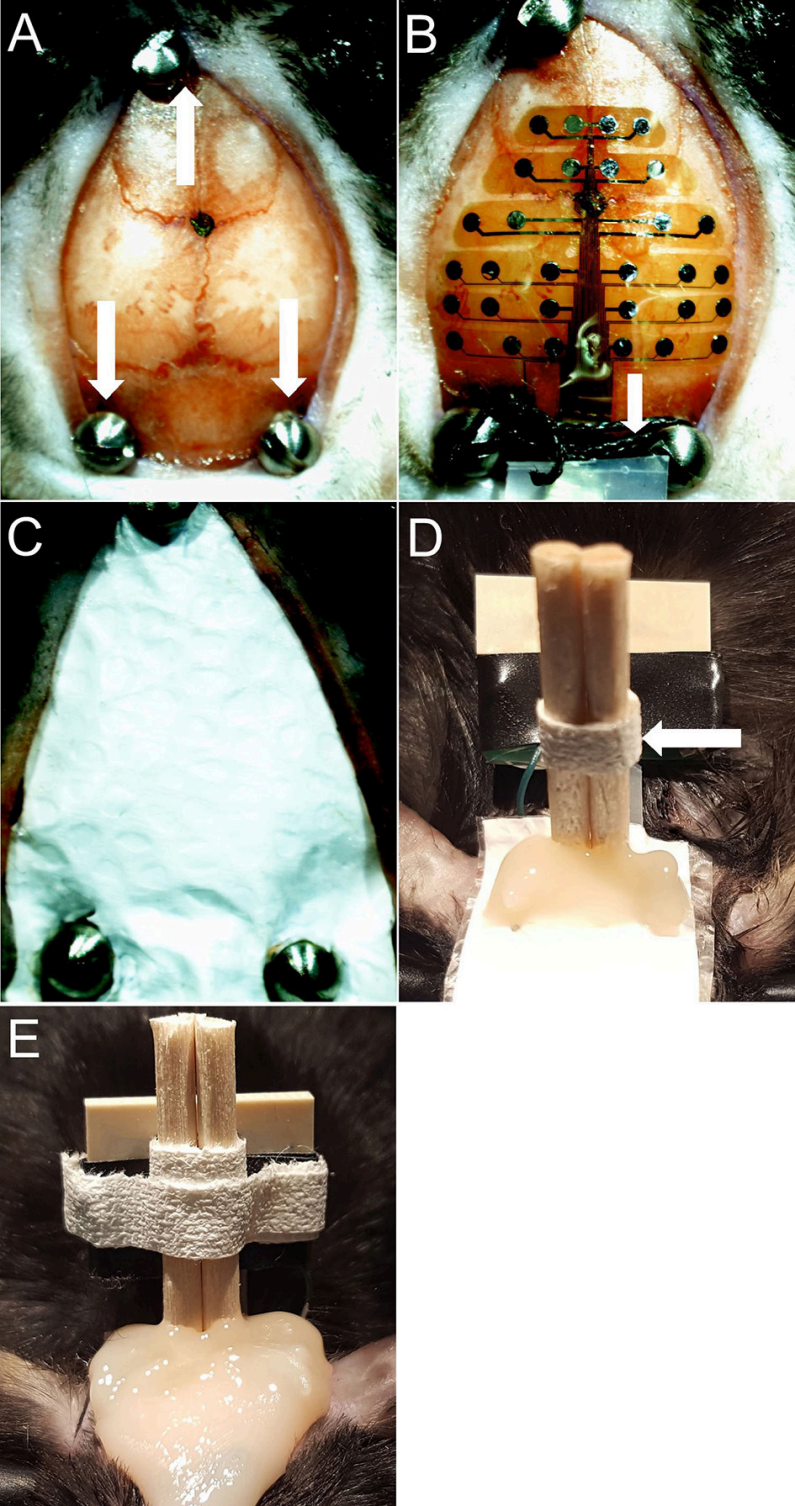
**(A)** Bregma marked with screws in place. White arrows indicate screws. **(B)** Probe dried on skull with saline. White arrows indicate silk tie. **(C)** Teflon & saran wrap (MEA protective layer). **(D)** Dental cement applied to secure the cotton-tip applicator and back screws. White arrows indicate cotton-tip applicator pieces secured with medical tape. **(E)** Final picture.

1. Anesthetize mice with isoflurane inhalation (0.2–0.5%) and administer ketamine/xylazine (80/10 mg/kg, i.p.). Remove hair with depilatory cream and prepare surgical site.
2. Load mouse into stereotactic frame. Make a midline sagittal incision along the scalp to expose the skull. Use cotton-tip applicator to remove periosteum from the skull and clean skull with saline.
3. Mark bregma and positions for 3 screws. Drill 1 mm diameter holes for the three skull screws (PlasticsOne, 00-96 X 1/16). Apply screws to skull taking care not to penetrate the dura (Figure 2A).
4. Place grounding wire into nuchal musculature.
5. Place MEA probe onto the skull surface and add a drop of saline on top of the probe. Carefully align the “+” in the center of the probe with bregma (Figure 2B). Use PVA surgical spears (Braintree, SP 40815) to blot and remove excess saline from the probe. The probe surface does not have any adhesive; therefore, the saline allows the probe to adhere to the skull once it dries. The precise position of the MEA probe can be adjusted slightly before it dries; however, if the probe needs to be repositioned a drop of saline must be reapplied to the probe to avoid tearing it.
6. Tie a 4-0 silk tie between the two rear screws and slide down on top of the MEA ribbon to hold it in place.
7. Add Teflon and then saran wrap on top of probe (as protective layers for the MEA), cut to fit over the entire probe. Make 3 small holes in the Teflon/saran wrap layer to fit over the screws (Figure 2C).
8. Add dental cement (Kuraray, 3382KA) to the two back screws securing the MEA ribbon further in place. Cure with UV light.
9. Cut the wooden ends of cotton-tip applicator into two pieces, about 2 cm in length for each. Tape the two pieces together with waterproof medical tape (Kendall, 3142C). Place them in a vertical position between the two back screws and add dental cement to hold it in place (Figure 2D).
10. Use waterproof medical tape to secure the cotton-tip applicator pieces to the probe connector. This serves as an anchoring “post” for the probe connector to ensure that it is in a fixed upright position while the mouse is moving around during recording.
11. Apply final layer of dental cement over frontal screw and Teflon/saran wrap layer (Figure 2E).
12. Apply triple antibiotic ointment to the edges of the dental cement. Administer buprenorphine (0.1 mg/kg, s.c.) for postoperative pain control. Allow mouse to recover for a minimum of 2 days prior to electrophysiological recording.

### *In vivo* mouse MEA EEG recording

**Figure 3:**
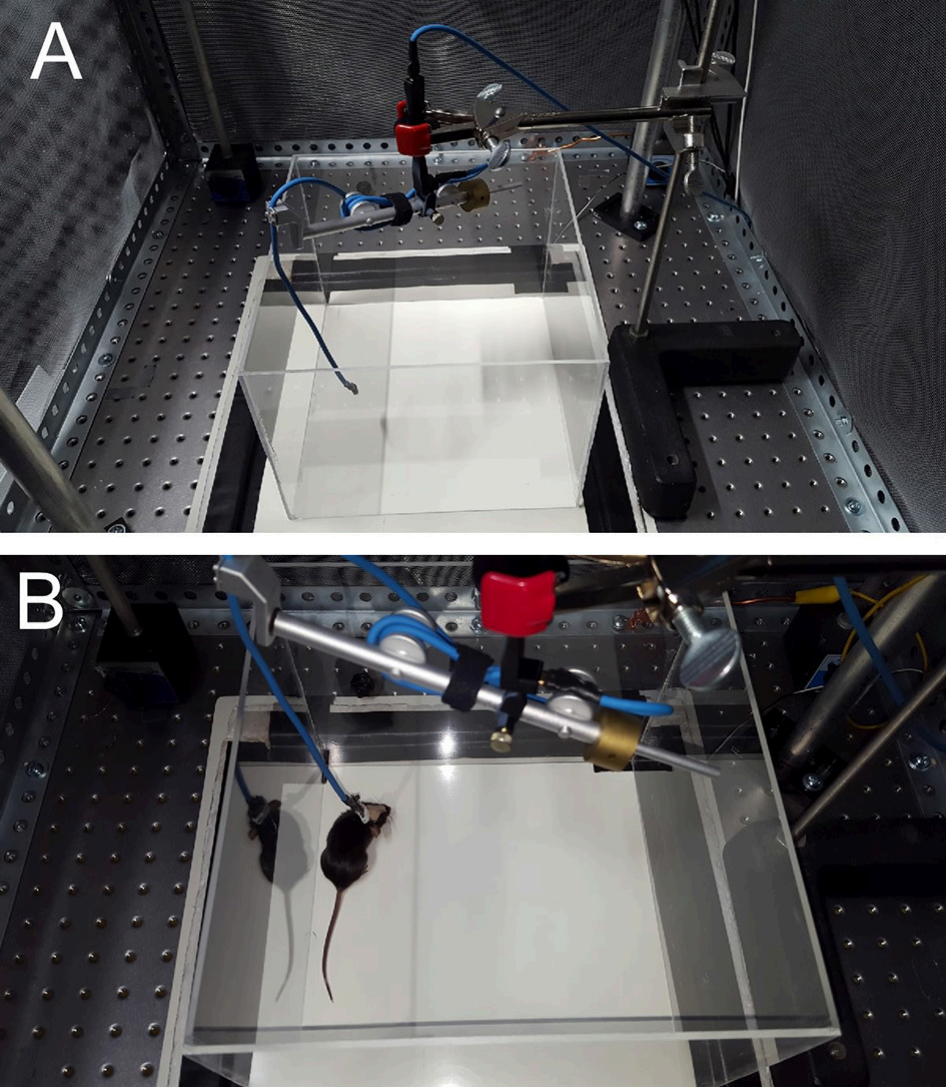
**(A)** Commutator set-up. **(B)** Still image of animal in soundbooth.

1. On the day of electrophysiological recording, the mouse is transferred to an observation cage connected to the recording equipment. The observation cage is protected by a Faraday cage for electrical shielding.
2. Set up the commutator (Figure 3A). The commutator (NeuroNexus) is designed to allow free movement of the mouse and the cables without restriction. A counterbalance arm maintains variable tension on the cable to prevent coiling as the animal moves throughout the cage. We have found that using a ring stand to hold the commutator works well.
3. Administer isoflurane briefly to the mouse and connect to the headstage and commutator.
4. Once the mouse is connected to the recording apparatus (Figure 3B), allow about 15 min of habituation to the cage and commutator.
5. Stimulus presentation and MEA recordings can now be initiated. The system we describe uses NeuroNexus MEA probes connected through the headstage to a NeuroNexus SmartBox™ amplifier and then multichannel digital EEG is stored by computer. However, this overall implantation protocol could be modified for any skull surface MEA probe configuration.

### Acoustic stimulation

All experiments were conducted in a sound-attenuated chamber lined with anechoic foam (Gretch-Ken Industries, Oregon). Acoustic stimuli were generated using RVPDX software and RZ6 hardware (Tucker Davis Technologies, FL) and presented through a free-field speaker (LCY-K100 ribbon tweeters; Madisound, WI) located 12 inches away directly above the cage. Sound pressure level (SPL) was modified using programmable attenuators in the RZ6 system. Speaker output was ~70 dB SPL at the floor of the recording chamber with fluctuation of ±3 dB for frequencies between 5 and 35 kHz as measured with a ¼ inch Bruel & Kjaer microphone. Sound delivery was synchronized with EEG recording using a TTL pulse to mark the onset of each sound in a train. Five minutes of resting EEG was recorded in which no auditory stimuli were presented. This was followed by ERP recordings in response to trains of broadband noise, and chirp stimuli.

### Synchronization indexing methods

To measure synchronization and determine the presence of volume conduction using the 30-channel MEA probe, two indices were calculated as has been done previously using MEA in rats (Stienen et al., 2016). Data used for calculation were collected from 5 mice during a 5-min period of wakefulness in the absence of auditory stimulation. Each 5 min of data was segmented into 1-s windows (~followed by FFT from 1 to 100 Hz using 1 Hz resolution. Complex numbers derived from the FFT were used to calculate synchrony. Phase Locking Value (PLV) is defined as:

$$\begin{array}{l} PLV=\frac{1}{N}|\overset{N}{\underset{n=1}{\sum }}\exp\left(j\theta \left(n\right)\right)| \end{array}$$

where *n* = trial and θ(*n*) is the phase difference between two channels θ_1_(*n*)–θ_2_(*n*) (Lachaux et al., 1999). This is a measure of inter-trial variability of phase difference between channels and was calculated for all unique pairs of 30 channels and for each frequency from 1 to 100 Hz. In addition, the Weighted Phase-Lag Index (WPLI), another index of synchrony was calculated because it minimizes the effects of volume conduction. WPLI (ϕ) is defined as:

$$\begin{array}{l} \phi \equiv \frac{\left|E\left\{ℑ\left\{X\right\}\right\}\right|}{E\left\{\left|ℑ\left\{X\right\}\right|\right\}} \end{array}$$

Where ℑ{*X*} is the imaginary components of the cross spectrum between 2 channels. Using this approach minimizes the effects of volume conduction compared to PLV by only considering the imaginary part and through normalization (Vinck et al., 2011).

## Results

**Figure 4:**
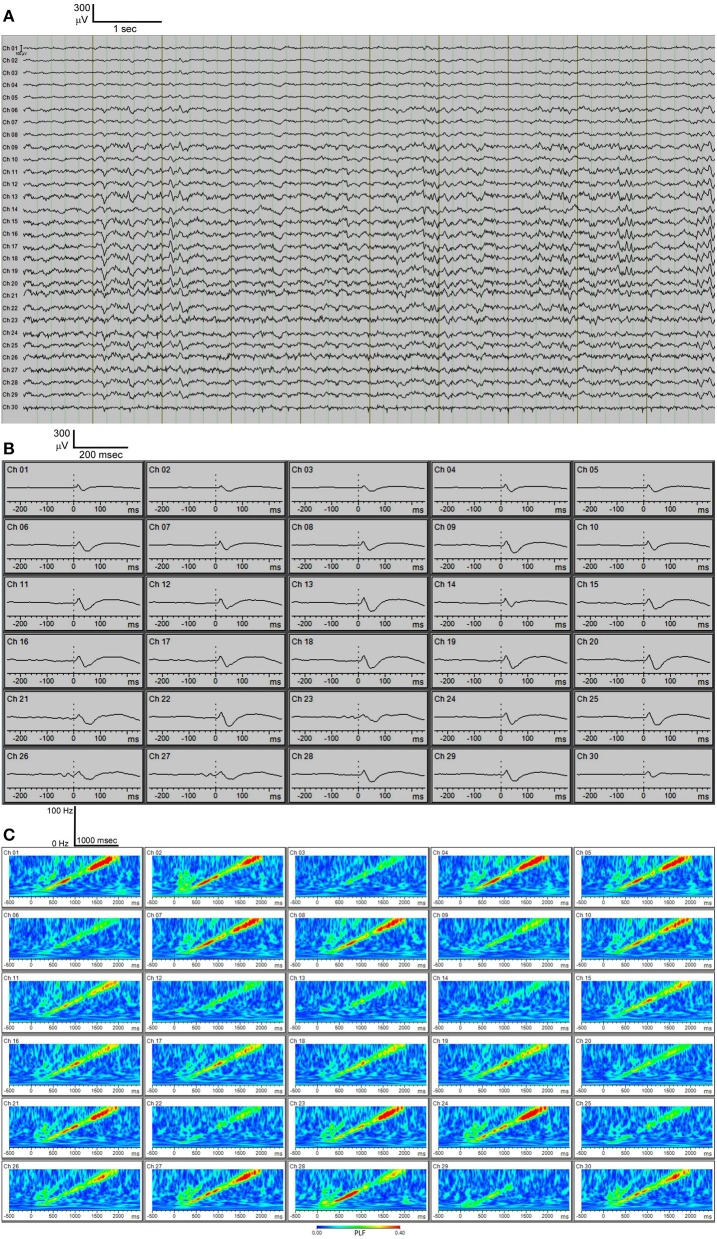
**(A)** C57BL/6 30-channel baseline EEG. **(B)** C57BL/6 30-channel auditory event-related potentials (ERPs) showing typical ERP waveforms. **(C)** C57BL/6 phase-locking factor (PLF) of EEG response to “up chirp” auditory stimulus.

Using the above methodology, we have reproducibly acquired multichannel EEG recordings (Figure 4A). In our system, we have an auditory stimulus protocol with either broadband noise or chirp stimuli. Broadband noise reliably elicits auditory event-related potentials (ERPs) using this system with low noise (Figure 4B). The chirp is a broadband noise stimulus whose amplitude is modulated 100% by a sinusoid with the frequency of modulation increasing or decreasing from 1 to 100 Hz. Chirp stimuli induce phase-locked EEG responses in the mouse that can be shown using “phase-locking factor” (to obtain inter-trial coherence) (Figure 4C) just as in data from auditory chirp stimuli applied to FXS human subjects (Ethridge et al., 2017).

EEG recordings can be collected from awake, freely moving mice in the cage attached to the commutator (Supplementary Video) for prolonged periods of time (several hours). Repeated recordings can be obtained from the same mouse days later. Interpretation of the EEG recordings obtained via the MEA is aided by careful evaluation of the probe map. This information can be analyzed by a variety of EEG analysis programs, such as BrainVision Analyzer 2. Derived EEG parameters can include: baseline EEG power, stimulus-evoked EEG power, EEG phase synchronization, wavelet analysis, cross-frequency coupling such as theta-gamma coupling, functional connectivity analysis, and heat map analysis.

**Figure 5:**
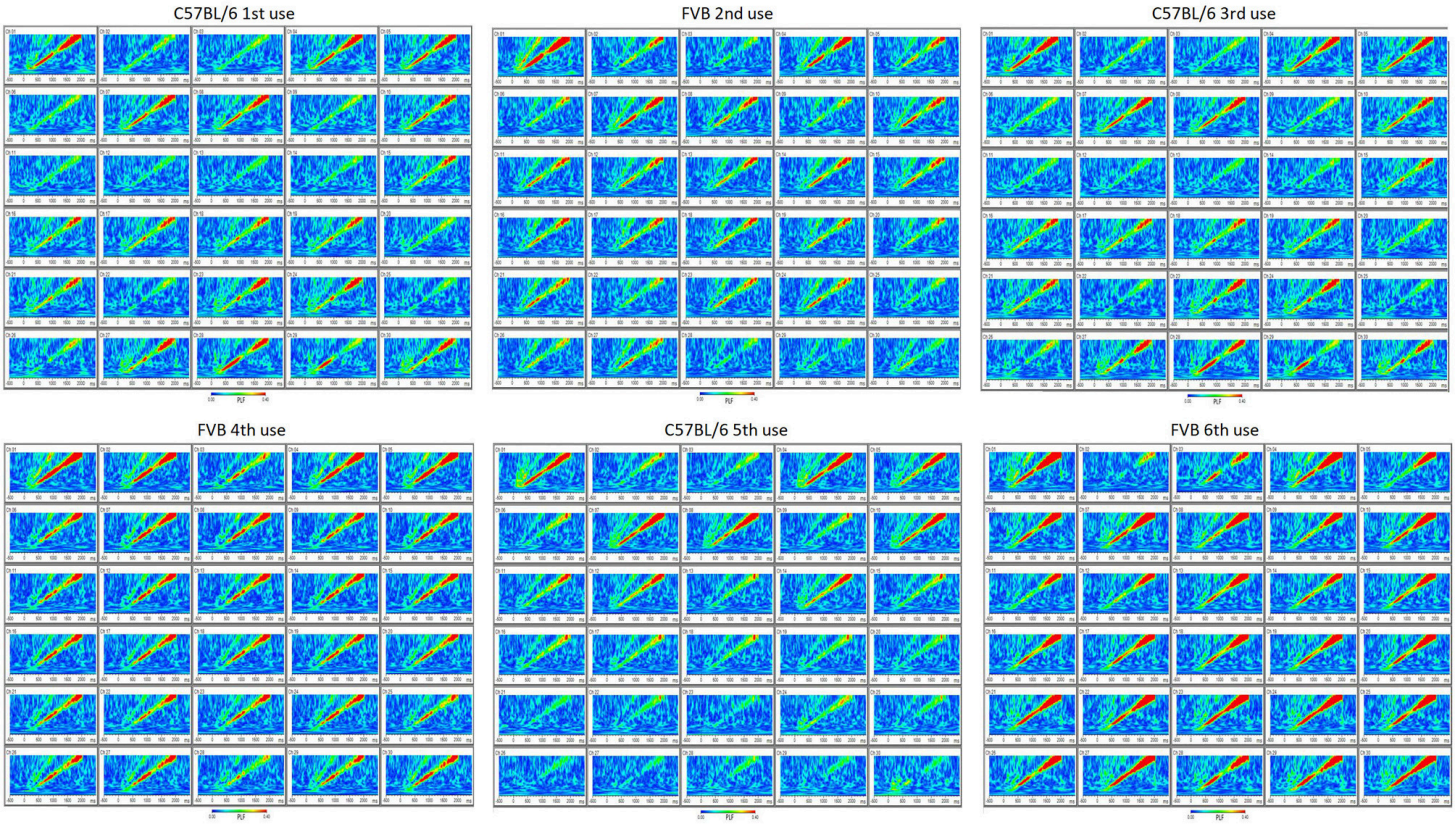
Reusability of probe (up to six uses) is demonstrated by reproducibility of phase-locking factor (PLF) of EEG response to “up chirp” auditory stimulus from C57BL/6 and FVB mouse strains.

Furthermore, the MEA probe can be removed and reused using our protocol. Following sacrifice and removal of the implant, the MEA probe can be carefully peeled off undamaged from under the Teflon protective layer, cleaned with ethanol and stored for re-use. Using this protocol, we have been able to reuse these MEA probes up to 6 times with no reduction in EEG quality (Figure 5). In addition, at each reuse we have measured electrode impedances and these values have remained within the normal range set by the manufacturer for every channel (data not shown). This reusability makes the entire enterprise of *in vivo* MEA more efficient and economical.

**Figure 6:**
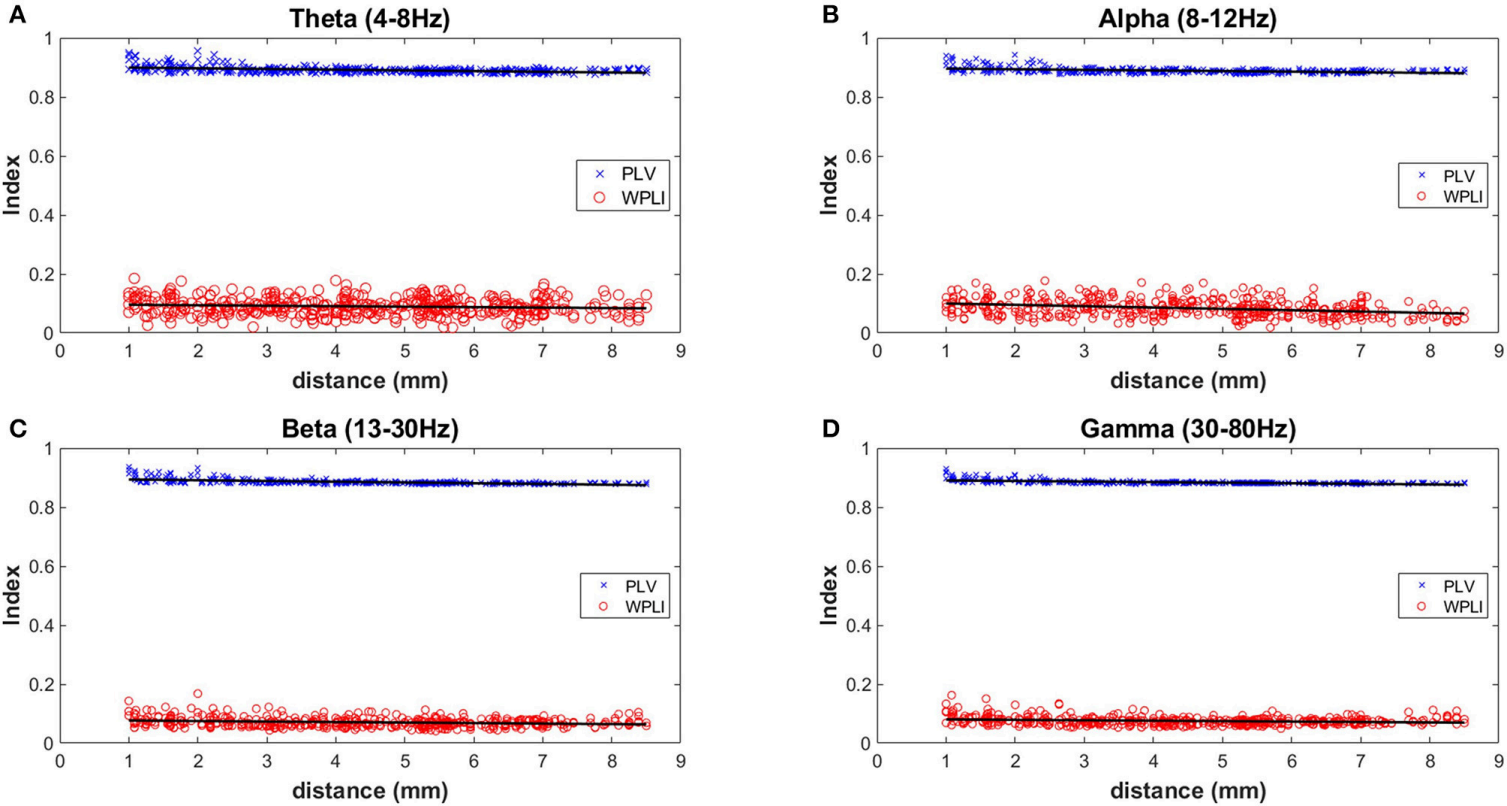
Measures of inter-electrode synchrony across frequency bands and distance. Two measures of synchrony were calculated for each unique electrode pair (435 pairs) across 4 different frequency bands during wakefulness (*n* = 5 mice). Analysis was done on 4 different frequency bands and the mean values of all mice are plotted in **(A)** Theta (4–8 Hz), **(B)** Alpha (8–12 Hz), **(C)** Beta (13–30 Hz), and **(D)** Gamma (30–80 Hz).

Two measures of synchrony were calculated for each unique electrode pair (435 pairs) across 4 different frequency bands during wakefulness (n = 5 mice) (Figure 6). The PLV (Phase Locking Value) index is known to be sensitive to the effects of volume conduction, while the WPLI (Weighted Phase-Lag Index) is designed to minimize the effects of volume conduction. Therefore, the separation of the PLV vs. WPLI is an approach to gauge the degree of volume conduction present in recordings {Stienen, 2016 #26}. The largest distance measurable on the mouse skull using the MEA is 8.5 mm and shortest is 1.0 mm. All indexes showed a very slight downward slope (from −0.0014 to −0.0045) with increasing distance (Figure 6). A large separation between the PLV (~0.9) and WPLI (~0.1) was consistently observed at the 4 different frequency bands measured. This result is indicative of the presence of volume conduction using these measures of cross-channel synchronization. Despite this, channel-wise differences can still be observed in both auditory evoked ERPs (Figure 4B) as well as phase-locking to chirp stimulation (Figure 4C).

## Discussion

In this report, we describe a method for stable chronic *in vivo* implantation of a multielectrode array (MEA) on the surface of the mouse skull. This can then be used for baseline and stimulus-evoked EEG acquisition in awake, freely moving mice. Features of our protocol include: (1) standardized implantation procedure; (2) reproducible placement of probe over the skull surface with bregma as reference; (3) Teflon/saran wrap protective layer for the MEA probe to enable reusability; (4) secure implantation with dental cement and screw fixation; (5) fixation of the headstage with an anchoring “post”; (6) use of commutator to allow free movement of the mouse and cables without restriction; and (7) reproducible artifact-free 30-channel EEG. Of course, we encourage investigators to modify our protocol as needed to accommodate any desired skull surface MEA configuration for a particular application.

Only a few previous studies have attempted to obtain multisite EEG from mouse scalp or skull. In 2010, Choi et al. described a method for skull surface MEA recording in which the MEA was affixed with dental cement; in this paper, they demonstrated that high density EEG could help with source localization of seizure activity in an absence seizure model (Choi et al., 2010). This system has recently been applied to spatiotemporal evaluation of neuronal oscillations during REM sleep (Kim B. et al., 2017). Wasilczuk et al. constructed a 26-channel epidural array (placed through burr holes onto the dura) for acquisition of multisite mouse EEG and applied it to spatial evaluation of visual evoked potentials (Wasilczuk et al., 2016). A recent paper describes a method of 16-channel scalp EEG in mice and the ability to record visual evoked potentials, but the mice were fixed in a stereotactic frame and not freely moving (Kim D. et al., 2017). Our protocol is an improvement over the existing published protocols in that (1) these particular MEA electrodes are available commercially in a variety of configurations; (2) the MEA can be chronically implanted in order to record EEG in freely moving mice; (3) the MEA electrodes can be reused thus significantly reducing supply expenses for animal studies. Our goal of providing this detailed protocol is to distill over 1 year of constant effort of protocol development aimed at optimizing preparation time and surgical reproducibility. Thus, faithful recapitulation of this protocol will enable those with existing skills in stereotactic surgery to expand their repertoire to implement MEA in their laboratories.

Application of our MEA skull surface probe implantation technology will accommodate translational relevance of EEG biomarkers in rodent models of human neurological and neuropsychiatric disorders. For example, characteristic EEG abnormalities have been observed in Fragile X syndrome (Castrén et al., 2003; Van der Molen and Van der Molen, 2013; van der Molen et al., 2014; Ethridge et al., 2016, 2017; Wang et al., 2017), schizophrenia (Fejgin et al., 2014; Light and Swerdlow, 2015; Dvey-Aharon et al., 2017; Gomez-Pilar et al., 2017) and Rett syndrome (Buoni et al., 2008, 2010; Liao et al., 2012; Pini et al., 2016). Thus, if rodent EEG studies of models of these disorders display electrophysiological features similar to human subjects, that could lead to validation of EEG biomarkers in rodents that can be used in pre-clinical studies (Sinclair et al., 2017). For example, EEG studies in rodent models of FXS have characterized abnormalities in baseline and stimulus-evoked EEG recordings. A recent EEG study of *Fmr1* KO rats demonstrated abnormal hyperactivity of the visual cortex during quiet rest, and reduced synchronization between fast-spiking interneurons (Berzhanskaya et al., 2017). *Fmr1* KO mice were found to have reduced habituation of the auditory ERP N1 amplitude as in humans with FXS; and this habituation could be rescued with deletion of matrix metalloproteinase MMP-9 (Lovelace et al., 2016). This study, done with one electrode placed in the auditory cortex, suggested that ERP habituation might serve as an “EEG biomarker” for FXS. Proof of principle for using ERP phenotypes as a disease biomarker comes from evidence that minocycline treatment was able to normalize auditory ERP amplitudes and habituation in children with FXS (Schneider et al., 2013). The authors conclude that ERPs may be useful as a biomarker in FXS.

Multielectrode EEG recording (30-channel in our case, but other multichannel probes could be substituted with distinct numbers of electrodes) enabled by the skull-surface MEA technique will likely also enable better anatomic segregation of EEG contributions from discrete brain areas, and will enable complex EEG analysis. Studies with skull screw electrodes usually are limited to one or several active electrodes, often just unilateral, distinct from the 30-electrode configuration we have implemented here which covers wide areas of the frontal, temporal and parietal areas bilaterally. Various types of EEG analysis are feasible on the derived MEA data (Roach and Mathalon, 2008). In addition to standard spectral analysis of resting state EEG signals, analysis of stimulus-evoked EEG responses (such as auditory ERPs) is also feasible. As shown in Figure 4B, we can obtain distinct auditory ERP data from each of the 30 channels separately. The probe map can be used to make groupings of nearby electrodes, for example to consolidate areas of the MEA probe into left frontal, right frontal, left temporal, right temporal, left medial, and right medial regions (data not shown). Finally, if desired, all 30 channels can be “collapsed” into one channel to examine average EEG signals across all 30 channels. Other derived data available from EEG amplitude information include stimulus-locked EEG power and non-stimulus-locked EEG power. From EEG phase data we can obtain “phase-locking factor” time/frequency plots as shown in Figure 4C. Such phase data can also be consolidated into 6 areas or 1 overall “average” channel. Thus, EEG amplitude and phase data can be fully analyzed across widespread areas of cortex. It is important to be aware that a limitation of EEG with MEA might be volume conductance (Stienen et al., 2016). We observed, like Stienen et al. (2016), that volume conductance can be measured (Figure 6) but nevertheless channel-wise differences can still be observed in both auditory ERPs (Figure 4B) as well as phase-locking to chirp stimulation (Figure 4C).

Furthermore, computation of coupling of various EEG bands across different brain areas is feasible with the MEA data. Interestingly, humans with FXS have been shown to have altered theta-gamma coupling (Ethridge et al., 2017; Wang et al., 2017); our MEA technology now allows detailed assessment of theta-gamma coupling across the surface of the brain in *Fmr1* KO mice. Other EEG analyses that can be performed on multichannel MEA data include wavelet analysis (Ethridge et al., 2017), coherence, and principal component analysis, which can be used to address questions of functional connectivity and interaction across cortical regions. Combination of ERP measures in distinct genetic models can also reveal the cellular source of auditory ERP deficits as has been shown in Rett syndrome (Goffin et al., 2014). Finally, this technology combined with acute and chronic drug studies will allow for detailed evaluation of pharmacological alterations in EEG signals in various mouse models of disease (Cambiaghi et al., 2013).

In summary, we have implemented skull surface MEA *in vivo* in awake, freely moving mice which enables chronic multisite EEG recording and analysis. Furthermore, we demonstrate reusability of the MEA probes, which is economically critical for experiments with many animals. The increased use of scalp MEA type recordings by the neuroscientific community will facilitate translation-relevant research platforms across various neurophysiological and pathophysiological contexts.

## Author contributions

DB helped conceive and design the experiments and analyze the data in this manuscript and co-wrote the manuscript. CJ designed and performed all of the experiments and analyzed data in this manuscript and co-wrote the manuscript. JL helped with data analysis. IE and KR helped conceive of the experiments and co-edited the manuscript.

### Conflict of interest statement

The authors declare that the research was conducted in the absence of any commercial or financial relationships that could be construed as a potential conflict of interest.

## Supplementary material

Supplementary Video Freely moving mouse in recording cage with MEA in place.

## Abbreviations

ECoG: electrocorticography
EEG: electroencephalography
LFP: local field potentials
MEA: multielectrode array.
